# Application of chitosan nanopriming on plant growth and secondary metabolites of *Pancratium maritimum* L

**DOI:** 10.1186/s12870-024-05148-8

**Published:** 2024-05-28

**Authors:** Eman Allam, Salama El-Darier, Zekry Ghattass, Amal Fakhry, Roufaida M. Elghobashy

**Affiliations:** 1 Institute of Graduate Studies & Research, Alexandria University, Alexandria, Egypt; 2 Botany and Microbiology Department, Faculty of Science, Alexandria University, Alexandria, Egypt; 3 Biology and Geology Department, Faculty of Education, Alexandria University, Alexandria, Egypt

**Keywords:** Biostimulants, Medicinal plants, Nanoparticles and plant biochemistry

## Abstract

**Background:**

Nanotechnology has demonstrated its vital significance in all aspects of daily life. Our research was conducted to estimate the potential of primed seed with chitosan nanoparticles in seed growth and yield by inducing plant secondary metabolism of *Pancratium maritimum* L. one of the important medicinal plants. Petri dish and pot experiments were carried out. Seeds of *Pancratium maritimum* L. were soaked in Nano solution (0.1, 0.5, 1 mg/ ml) for 4, 8, 12 h. Germination parameters (germination percentage, germination velocity, speed of germination, germination energy, germination index, mean germination time, seedling shoot and root length, shoot root ratio, seedling vigor index, plant biomass and water content), alkaloids and antioxidant activity of *Pancratium maritimum* L. were recorded and compared between coated and uncoated seeds.

**Results:**

Our results exhibited that chitosan nanopriming had a positive effect on some growth parameters, while it fluctuated on others. However, the data showed that most germination parameters were significantly affected in coated seeds compared to uncoated seeds. GC-MS analysis of *Pancratium maritimum* L. with different nanopriming treatments showed that the quantity of alkaloids decreased, but the amount of pancratistatin, lycorine and antioxidant content increased compared with the control.

**Conclusions:**

Applying chitosan nanoparticles in priming seeds might be a simple and effective way to improve the quantity of secondary metabolites of *Pancratium maritimum* L. valuable medicinal plant.

## Introduction

*Pancratium maritimum* L. is a plant of ecological and medicinal importance, a perennial bulbous plant from Amaryllidaceae family. which thrives in coastal settings and has unique physiological characteristics that allow it to tolerate adverse environmental circumstances such as excessive salt and drought. Furthermore, it is well-known for its diverse repertoire of secondary metabolites, Previous studies on *P. maritimum* have revealed the presence of various alkaloids. These studies have also shown that the alkaloids and flavonoids found in the plant’s bulbs possess pharmaceutical properties [1–4]. Specifically, pancratistatin and lycorine, *P. maritimum* has anti-bacterial, anti-malarial, purgative, anti-viral, immune-stimulant, analgic, anti-cancer, antifungal, and antioxidant activities [1, 5]. Sustainable use of botanical medicines is crucial, not only for their importance as a possible source of new pharmaceuticals, but also for the dependence on traditional herbal remedies for health [6]. Secondary plant metabolites were used in traditional medicine and folklore to treat a variety of diseases. For many years, researchers have been looking into strategies to improve crop species growth and development. Few research studies dealing with the enhancement of medicinal plant seeds in this aspect are available. in order to improve the growth of the plant and maximize the production of these metabolites, researchers have been investigating different strategies, and one particularly promising approach is nanopriming with chitosan.

In recent years, advances in agricultural science have seen a surge of interest in developing novel ways to boost plant growth and maximize secondary metabolite production in medicinal plants. Among these options, nanotechnology has emerged as a promising technology. Extensive research was recently undertaken on the interaction of nanoparticles with agriculture. Several approach have been developed, such as improving soil properties, improving germination, increasing uptake of nutrients, controlling pests, delivering genetic materials and overall plant performance [7].

Chitosan is a type of polymer formed by fungi, insect species, and crabs. It has antibacterial properties and positively affects the development and productivity of many plants [8]. Chitosan nanoparticles have excellent properties, as; biocompatibility, non-toxicity and Eco friendliness. In addition, chitosan has therapeutic properties such as inhibiting of the growth of microorganisms and promoting of cell growth [9].

Seed priming is a pre-sowing approach that influences seed germination and the growth of seedlings by altering pregermination metabolic processes prior to the appearance of the radicle. It generally improves the rate of germination and the performance of the plant [10]. Priming has an extensive effect on secondary metabolites, which range from simple sugars to complicated proteins. These metabolites play an important role in the germination potential. However, precise quality and quantity-based research is required to properly investigate all aspects of the alteration process involving priming.

The present study aimed to evaluate the effect of chitosan. nanopriming with different concentrations (0.1, 0.5, 1 mg/ ml) for 4, 8, and 12 h on coated and uncoated *P. maritimum* seeds on growth parameters. Furthermore, the study will look at the effect of chitosan nanopriming on the biosynthesis of secondary metabolites having pharmacological significance, with a focus on alkaloids, pancratistatin, lycorine and antioxidant activities.

## Materials and methods

### Plant materials

**Fig. 1:**
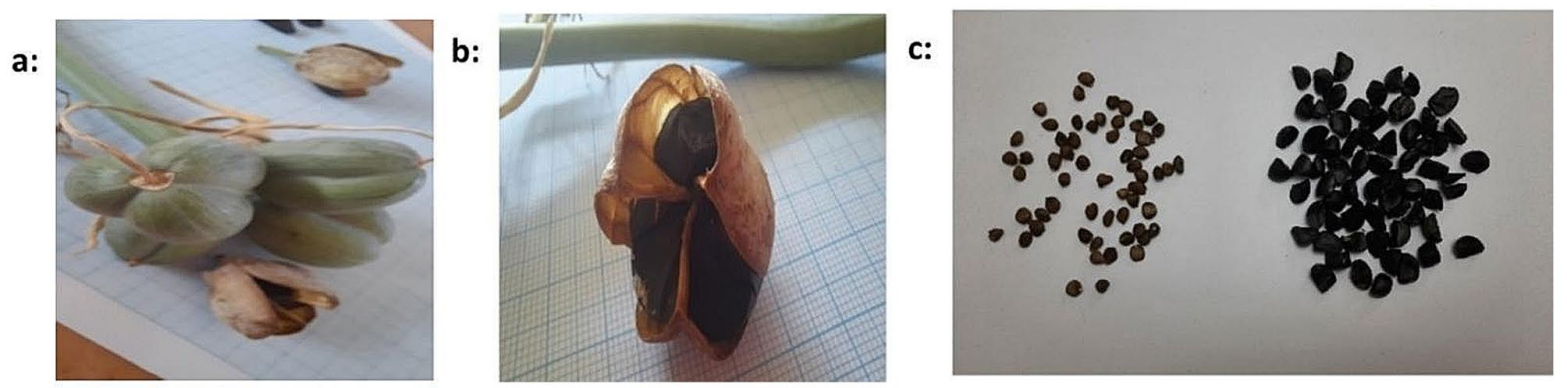
**a&b**: closed pods seeds of *Pancratium maritimum* L., **c**: coated and uncoated seeds of *Pancratium maritimum* L

Samples of mature seeds from closed pods of *P. maritimum* were collected in November 2022 (Flowering: July-September [11]), from Matrouh Governorate, on the Mediterranean coast, Egypt. Identification of plant materials was Performed by Prof. Dr. Salama El-Darier and confirmed by the Herbarium of the Faculty of Science, Alexandria University, the experiment was carried out at the Faculty of Science, Alexandria University, Egypt. The collected seeds were sorted into two groups coated seeds (with the auriferous parenchyma that covers them) and uncoated seeds (without the auriferous parenchyma that covers them) (Fig. 1). Both two groups of seeds were air-dried for 15 days at 25 °C, then stored in paper bags.

### Preparation of chitosan nanoparticles for nanopriming

Chitosan nanoparticles were purchased from NanoFab Technology Chemical Company, Giza, Egypt. Three distinct concentrations of chitosan nanoparticles (CsNPs) (0.1, 0.5, 1 mg/ ml) were used as priming solutions. The physical properties of the used chitosan nanoparticles have been taken from the attached pamphlet while its shape was defined by transmission electron microscope (JSM-1400 PLUS.JEOL, Japan).

### Preparation of seeds for laboratory experiments

Before soaking, the uniform seeds of coated and uncoated seeds of *P. maritimum* were sterilized for 2 to 3 min in a dilute solution of 3% sodium hypochlorite washed carfully four times with distilled water [12]. then left to dry in the air. Thirty seeds were imbibed in each concentration of chitosan nanoparticles (CsNPs) (0.1, 0.5, 1 mg/ ml) for 4, 8, and 12 h. The soaked seeds were rinsed with distilled water and then left in the air between two filter papers to dry back closer to the original moisture (12 h).

### Germination bioassay

Primed seeds are allowed to germinate in petri dishes (in Three replicates). Each dish contained 10 seeds grown on a no.1 Whatmann filter paper, irrigated with 20 ml of distilled water. A petri dish assay has been carried out in three replicates to evaluate the biological impact of chitosan nanopriming on the percentage of germination (GP), plumule (PL), and radicle (RL) lengths of *P. maritimum* and to compare coated and uncoated seeds. The experiment was performed under conventional laboratory conditions. with day temperatures ranging from 22-27^o^C and night temperatures from 14-18^o^C.

### Growth experiment

Primed and unprimed seeds were sowed in plastic pots (15 cm in width and 20 cm in length, with holes at the bottom). filled with a fixed amount of 1 kg of clean, sieved sandy soil, 10 seeds were planted separately in each pot at 0.5 cm depth from the soil surface. The pots were arranged in a completely randomized block design with 3 replicates. The amount of water corresponding to average soil-plant evapotranspiration calculated from weight loss over 24 h intervals. The experiment was performed under greenhouse conditions (20 ± 2ºC temperature, 75 ± 2% relative humidity, and 14/10 light/dark photoperiod).

When The plants became well established (after 99 days), The uniform seedlings were carefully picked from each treatment, cleaned with tap water to get rid of any remaining particles of soil, and then gently wiped with filter paper using distilled water. The samples were divided into roots and shoots to determine growth parameters. Dry weight was determined by drying other samples at 60 ºC until they reached a consistent weight.

### Growth parameters

The following equations were used to record some germination parameters,

**Germination percentage (GP%)** is calculated as the ratio of germinated seeds to total seeds multiplied by 100.

**Germination velocity (GVe) =** ∑ GP/ t where GP represents germination percentage and t represents total germination time.

**Speed of germination (SG) =** Ni/Di number/day [13] As *Ni* is the number of seeds germinated per day *Di*.

**Germination energy (GE)** is calculated by dividing the number of germinated seeds at 4 days by the total number of seeds tested × 100.

**Germination index (GI)** is equal to ∑ (Et / Dt), where Et is the number of emerging seeds in t days and Dt is the number of corresponding germination days.

**Mean germination time (MGT) =** Σfx/ (Σ (f1*x1) +………) where f is the number of seeds germinated on day x.

**Seedling shoot length (SL), Seedling root length (RL), Shoot Root ratio (SL/ RL ratio)** six plant individuals were used per treatment to measure shoot and root lengths with measuring tape. The shoot/root ratio was calculated for each treatment. **Seedling vigor index (SVI)** = Seedling total length (cm) x GP% as described by Hussain et al. [14]

### Determination of plant biomass and water content

Fifteen individuals were chosen from each treatment group, split into shoots and roots, and weighed individually. The samples were then oven-dried at 60 °C until they reached a consistent weight to calculate the biomass for each treatment. The water content percentage was estimated by the dry and fresh specimens for the shoot system of the analyzed species in the equation, expressed as.

$${\rm{WC}}\,{\rm{ \% }}\,{\rm{ = }}\,{\rm{ }}{\left( {{\rm{Fresh}}\,{\rm{ wt}}{\rm{. }}\,{\rm{-}}\,{\rm{ Dry}}\,{\rm{ wt}}{\rm{.}}} \right)_{\rm{*}}}\,{\rm{100 }}\,{\rm{/}}\,{\rm{ Fresh }}\,{\rm{wt}}{\rm{.}}$$

### Chemical analysis

#### Gas chromatography-mass spectrometry

The alkaloid extract of *P. maritimum* was obtained by extracting 100 mg of air-dried and powdered plant material with 1 mL methanol thrice. The nonpolar chemicals were eliminated with diethyl ether, and the residual acidic aqueous phases were basified with 25% ammonia. (25% NH_4_OH). Finally, the alkaloids were extracted with chloroform, dried with sodium sulfate, and vacuum distilled to obtain the alkaloidal extract [15].

The Thermo GC-Trace Ultra Ver: 2.0 and Thermo MS DSQ II in electron impact mode was utilized to analyze underivatized alkaloids. Methanol was used to dissolve the extracts, and analysis was carried out using a TR-5 MS column. Mass spectra were obtained in the m/z range of 50 to 450. The chemicals were identified by comparing their mass spectral fragmentation to previously isolated standards from Amaryllidaceae species.

### Antioxidant activity using DPPH radical method

Fresh plant samples were immersed in 80% methanol overnight, followed by filtration using Whatman No. 4 filter paper in preparation for antioxidant activity testing through the DPPH radical scavenging method [16].

Initially, 50 µl of plant extract at concentrations ranging from 10 to 100 µg/mL was combined with 2 ml of DPPH, and the mixture was then left in the dark at room temperature for 30 min. Meanwhile, a negative control involved 1 ml of methanol and 2 ml of DPPH, and a positive control utilized a methanol solution. Absorbance readings were taken at 517 nm. The calculation of the percentage of DPPH radical scavenging activity (% RSA) followed the formula:

%RSA = 100 _*_ (absorbance of control − absorbance of sample) / absorbance of control,

where the control consisted of 2 ml of DPPH and 1 ml of methanol.

The radical scavenging activities of the tested samples, expressed as percentage inhibition of DPPH, were calculated according to the following formula proposed by Molyneux in 2004 [17]:

$${\rm{A}}\,{\rm{ = }}\,{\rm{ }}\left( {{{\rm{A}}_{{\rm{control}}}}\, - {{\rm{A}}_{{\rm{sample}}}}} \right){\rm{ }}\,{\rm{/}}\,{\rm{ }}{{\rm{A}}_{{\rm{control}}}}_{\rm{*}}\,{\rm{100}}$$

where A_control_ is the absorbance at 515 nm of the blank sample at time t = 0 min, and A _sample_ is the final absorbance of the test sample at 515 nm.

### Statistical analysis

Descriptive statistics, including standard deviation (± SD), were employed to compare the differences among mean values. The data underwent analysis using IBM SPSS software package version 20.0 (Armonk, NY: IBM Corp). Quantitative data were characterized by mean and standard deviation. The significance of the results obtained was assessed at the 5% level. Statistical tests included the F-test (ANOVA) for normally distributed quantitative variables to compare more than two groups, and the Post Hoc test (Tukey) for pairwise comparisons.

## Results

**Fig. 2:**
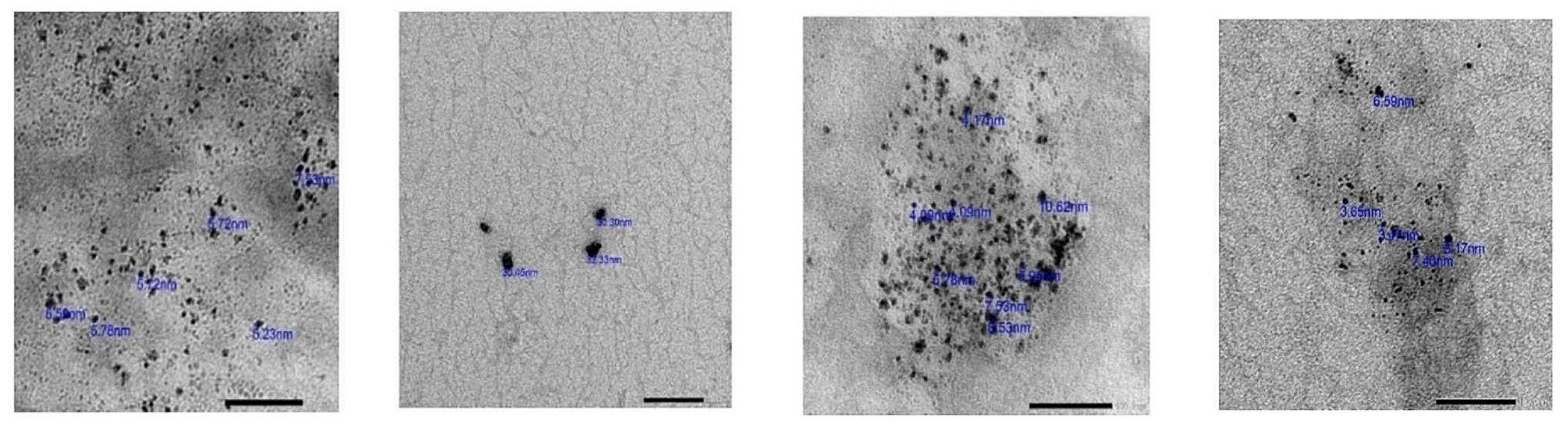
The shape and size of Chitosan nanoparticles seen by Transmission Electron Microscope (TEM).

The morphology of chitosan nanoparticles was examined using Transmission Electron Microscopy (TEM). The unmodified chitosan nanoparticles consisted of clusters of nanoparticles with sizes ranging from 3.6 to 32.33 nm and a spherical shape. This information is presented in Table (1) and Fig. (2).

**Table 1:** Characterizations of the prepared Chitosan nanoparticles

Synonyms:	Nanopoly (D-glucosamine); deacetylated chitin nanoparticles
Chemical Formula:	(C6H11NO4)n
Chemical Family:	Polymer
Appearance:	White powder.
Morphology:	Spherical.
Odour:	Odorless
Reactivity	Non-reactive
Chemical Stability	The product is completely stable.

### Petri dish bioassay experiment

**Fig. 3:**
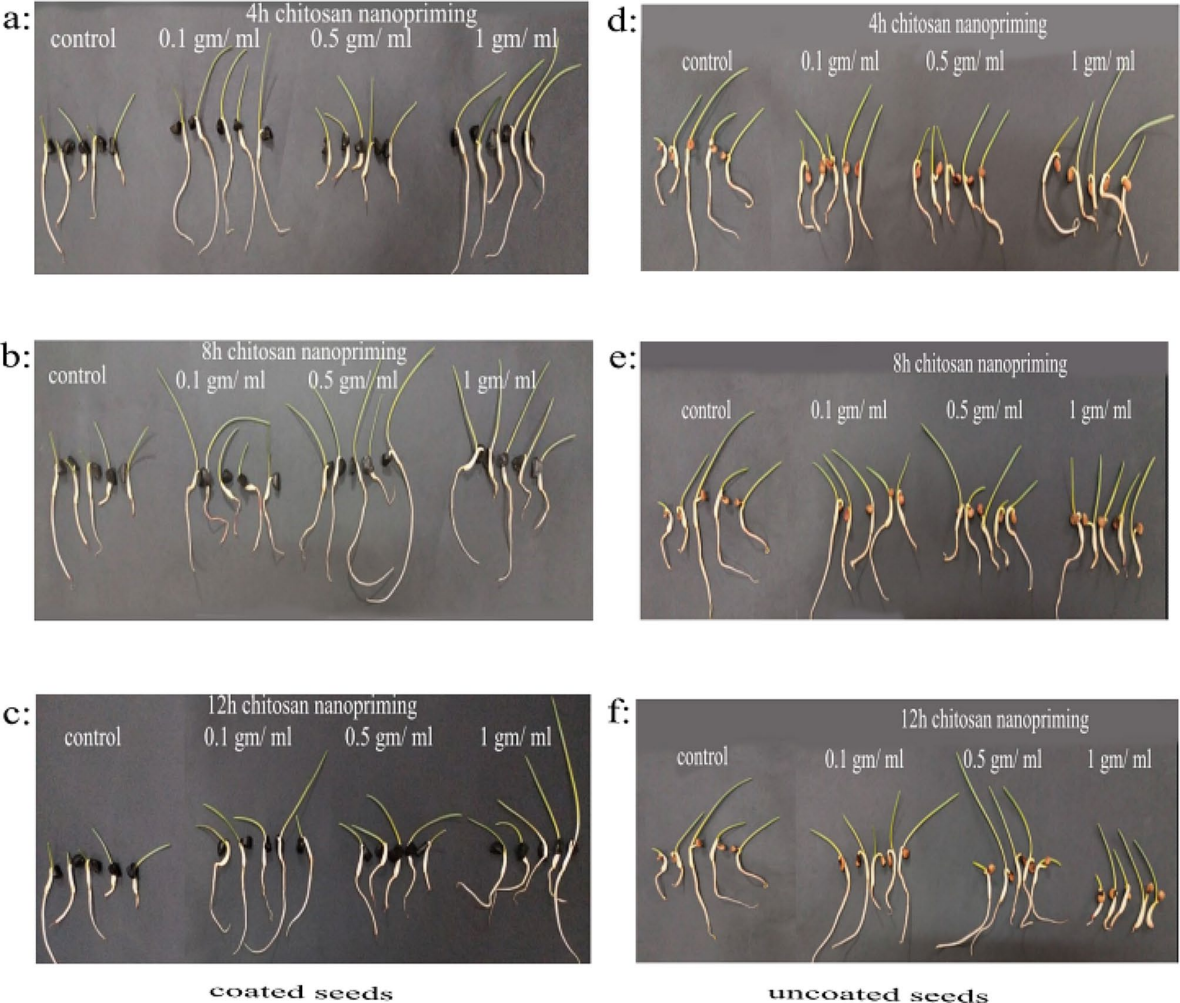
Effect of different concentration of nanopriming using chitosan nanoparticles (0.1, 0.5 and 1 mg/ml) on germination of coated (**a, b** & **c**) and uncoated (**d, e** and **f**) seeds of *Pancratium maritimum* L. in petri dish experiment under different soaking durations

**Fig. 4:**
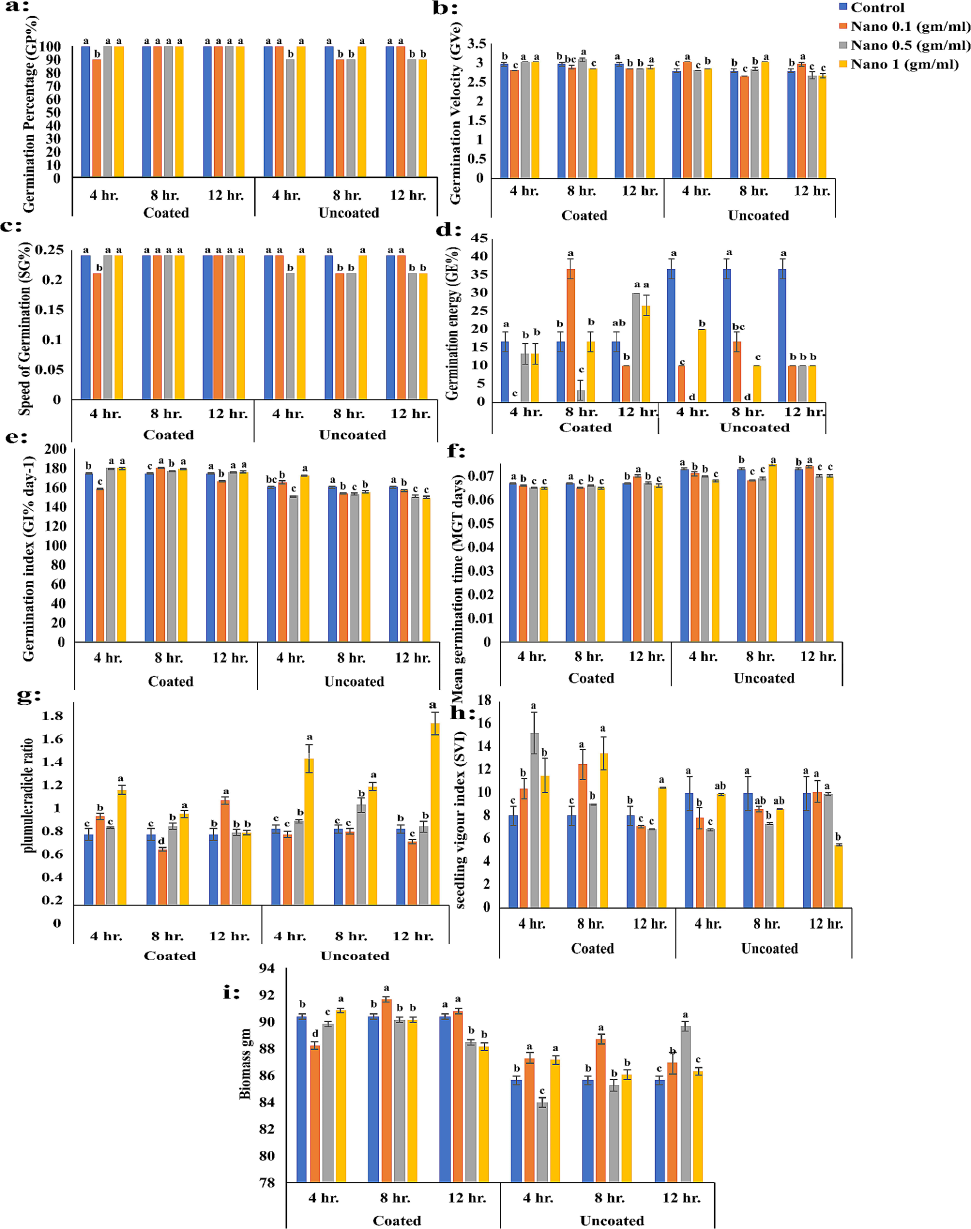
Variations of **a**: Germination Percentage (GP%), **b**: Germination Velocity (GVe). **c**: Speed of Germination (SG), **d**: Germination energy (GE), **e**: Germination index (GI), **f**: Mean germination time (MGT), **g**: plumule: radicle ratio, **h**: seedling vigor index (SVI**)** and **i**: Biomass in coated and uncoated seeds of *Pancratium maritimum* L. in petri dish experiment in response to different concentration of nanopriming under different soaking durations

The Petri dish experiment was conducted using chitosan nanoparticles as nanopriming with different concentrations (0.1, 0.5, 1 mg/ml), including unprimed seeds as a control. Each treatment had different soaking periods (4, 8, and 12 h). These treatments were assessed to evaluate some germination parameters of *P. maritimum* and compare the results of coated and uncoated seeds (Figs. 3 and 4). The results revealed that there were no significant variations in germination percentage (GP%) between nanopriming when compared to the control in both coated and uncoated seeds. However, there was a significant difference between coated and non-coated seeds, as the results showed that GP% was better in coated seeds (Fig. 4a).

In general, coated and uncoated seeds often had statistically significant differences in GVe at the control; it fluctuated in response to different priming techniques (Fig. 4b). No significant difference was observed in the speed of germination (SG) between coated and uncoated *P. maritimum* seeds after applying different seed priming treatments with varying durations of soaking (Fig. 4c). Several seed priming methods were found to cause a significant reduction in the germination energy (GE) of the chosen plant species when soaking uncoated seeds for 4, 8 and 12 h (Fig. 4d). Additionally, a noticeable increase in the germination index (GI) was observed in coated seeds after soaking for different durations. The highest significant value was achieved at the Nano 0.5 and 1 mg/ml treatment (Fig. 4e).

There was a significant decrease in the mean germination time (MGT) of nanopriming coated seeds. A decreasing trend was observed to reaching the highest reduction percentage (6.85%) compared to the control in response to the Nano 0.1 mg/ml treatment at 4 h and Nano 0.1 mg/ml treatment at 8 h (Fig. 4f). The plumule/radicle Ratio data showed a considerable increase after soaking for 4 h with Nano 1 mg/ml treatment, with the greatest percentages (62.42% relative to the control) being recorded (Fig. 4g). there was a significant increasing trend in plumule: radicle Ratio (139.17%) in uncoated seeds in response to the Nano 1 mg/ml treatment soaked for 12 h. although, the Nano 0.1 mg/ml treatment reduced the plumule/radicle Ratio by 16.62% when compared to the control.

In coated seeds soaked for 4 and 8 h, there was a highly significant increase in seedling vigor index (SVI). However, the recorded data showed that there is a distinguished decrease in seedling vigor index (SVI) in the case of 12 h soaking. It was observed that seedling vigor index (SVI) trends decreased noticeably after the uncoated seeds of *P. maritimum* were soaked for (8, and 12 h) (Fig. 4h). In general, the seedling vigor index (SVI) showed a statistically significant difference between coated seeds compared with uncoated seeds of *P. maritimum* in response to some seed priming treatments at different soaking periods. Several seed priming methods were found to cause significant variations in the biomass of the coated seeds of the selected plant species when soaking coated and uncoated seeds for different durations (Fig. 4i).

### Growth experiment

**Fig. 5:**
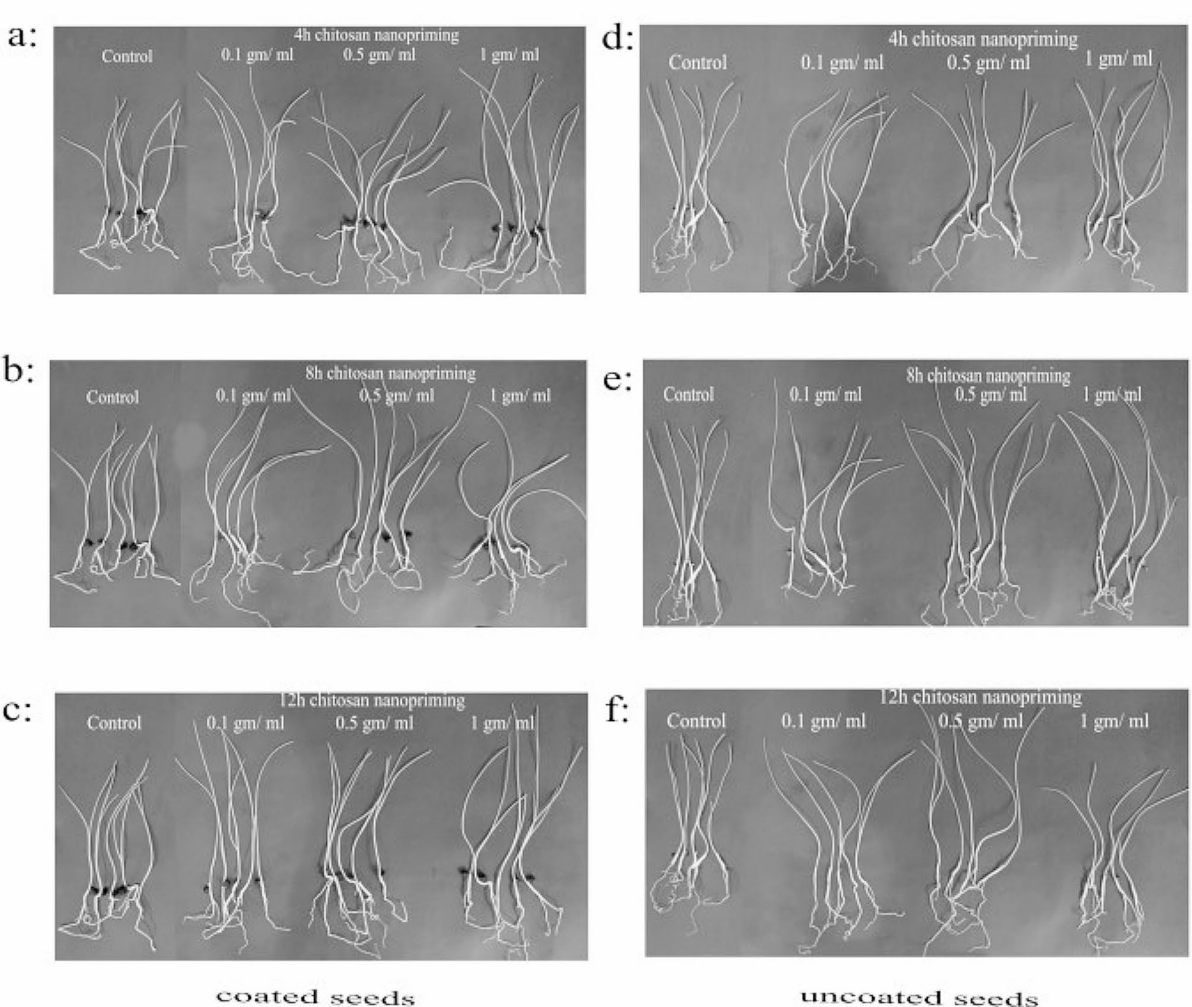
Effect of different concentration of nanopriming using chitosan nanoparticles (0.1, 0.5 and 1 mg/ml) on germination of coated (**a, b** & **c**) and uncoated (**d, e** and **f**) seeds of *Pancratium maritimum* L. in pot experiment under different soaking durations

**Fig. 6:**
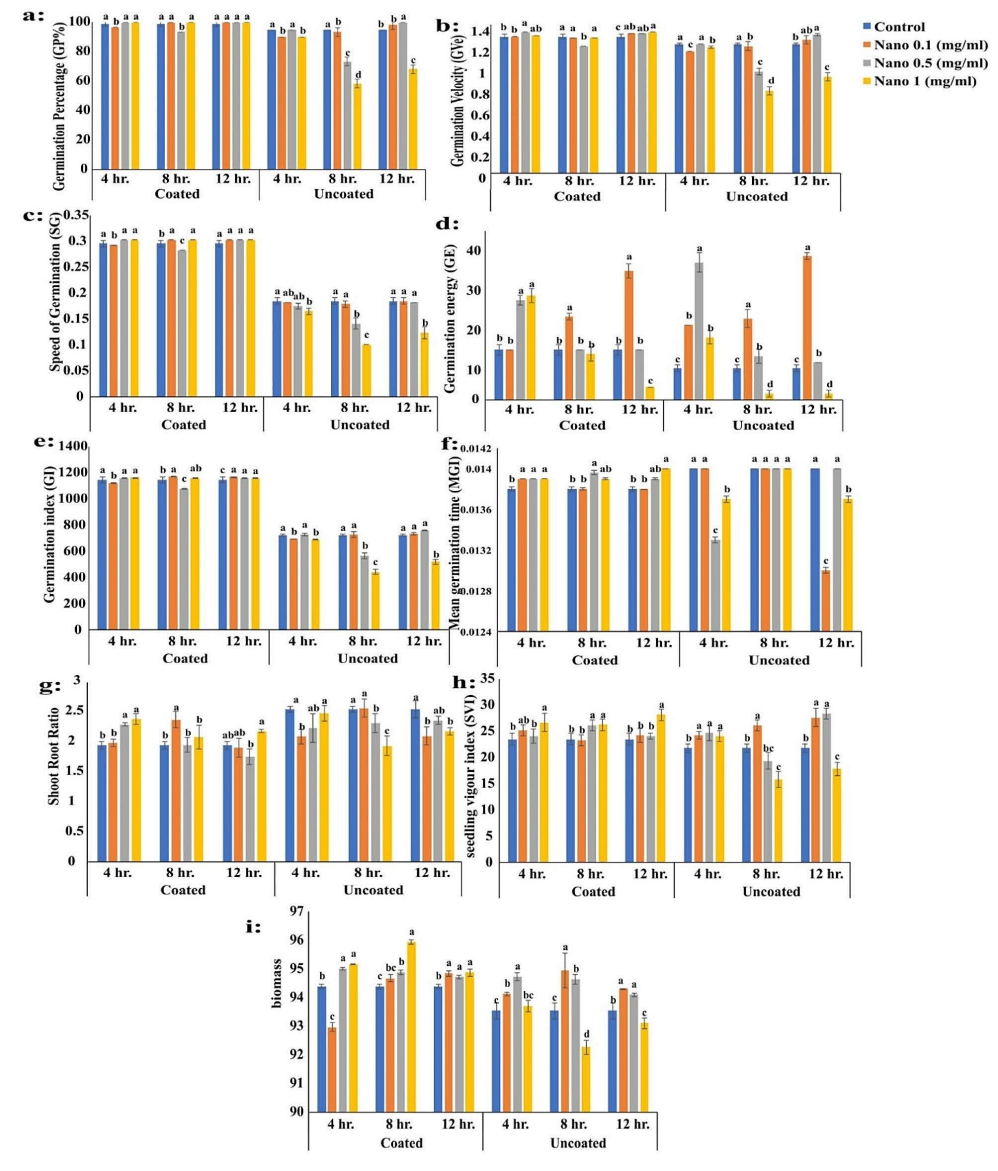
Variations of **a**: Germination Percentage (GP%),**b**: Germination Velocity (GVe). **c**: Speed of Germination (SG), **d**: Germination energy (GE), **e**: Germination index (GI), **f**: Mean germination time (MGT), **g**: shoot root ratio, **h**: seedling vigor index (SVI**)** and **i**: Biomass in coated and uncoated seeds of *Pancratium maritimum* L. in pot experiment in response to different concentration of nanopriming under different soaking durations

pots experiment was accomplished using nanopriming with chitosan nanoparticles in different concentrations (0.1, 0.5, 1 mg/ ml) beside unprimed seeds as control. The previously mentioned treatments used different soaking periods (4, 8 and 12 h) (Figs. 5 and 6). These treatments were assessed to evaluate some germination parameters of *P. maritimum* and compare coated and uncoated seeds. the coated seeds showed a higher GP% than the uncoated seeds of *P. maritimum*. However, there were no significant differences in GP% between nanopriming and the control in either case. The results for the coated seeds were found to be variable in GVe, but it was noted that data increased when the seeds were treated with 1 mg Nano after seed priming soaking for 12 h. Nano 0.5 mg/ml, and Nano 1 mg/ml for an 8-hour soaking period. Additionally, Nano 1 mg/ml for a 12-hour soaking period showed lower values of GVe in uncoated seeds compared to coated seeds.

Figure (6c) recorded Observations on SG of *P. maritimum* seeds, data revealed that there is a significant rise in data was detected in the case of coated seeds under various seed priming treatments for the three different soaking durations. This increase in SG is approximately 2.36% higher than the control. In contrast, data recorded a significant decrease in SG in individuals treated with Nano 1 mg/ml soaked in the three duration times. Nano 1 mg/ml treatment resulted in a significant decrease in SG Compared to the control, the decrease was 10.81%, 45.4%, and 32.97% respectively.

It was observed that the Nano treatment with a concentration of 0.1 mg/ml showed the most significant increase in GE after soaking for 8 and 12 h, with a percentage of 66.69% and 158.36%, respectively. However, when uncoated seeds were soaked for 8 and 12 h in response to Nano 1 mg/ml treatment, a significant decrease in GE was observed by the same percentage (79.95%) compared to the control (Fig. 6d). After soaking for 4 h, there was a noticeable increase in GI, with the highest percentage (1.42%) being recorded in response to Nano 1 mg/ml. After soaking for 12 h, the Nano treatment with 0.1 mg/ml had the highest value recorded (2.06% higher than the control). Overall, the various seed priming treatments were effective in producing a significant difference in GI between coated and uncoated seeds of *P. maritimum* (Fig. 6e).

the mean germination time (MGT) were displayed in the (Fig. 6f). data recorded a clear increase in MGT After the coated seeds of the plant species were soaked for 4 h,). It was observed that MGT trends increased noticeably after being soaked for 8 h. The treatment with Nano 0.1 mg/ml achieved the highest significant value, displaying a 2.17% increase compared to the control. After being soaked for 12 h, only the Nano treatment with a concentration of 0.1 mg/ml did not affect the mean germination time (MGT) when compared to the control. Other treatments showed an increasing trend in the MGT value by 0.72% compared to the control. soaking for 12 h with 0.5 mg/ml and 1 mg/ml Nano treatment resulted in a 0.72% and 1.45% increase in MGT, respectively when compared to the control. contrasty, when uncoated seeds of the chosen plant species were administered to different seed priming techniques at 4 and 12 h soaking durations, a decreasing trend in the MGI was observed to reach the highest reduction in MGT 7.69% compared to the control in response to Nano 0.1 mg/ml treatment at 12 h. The MGI was constant at 8 h in response to different Nano treatments.

The effect of different seed nanopriming methods at three different soaking durations (4, 8, and 12 h) on the Shoot Root Ratio in the case of coated and uncoated seeds of *P. maritimum* grown in pots were recorded in Fig. (6 g). There was a noticeable increase in trends for the Shoot Root Ratio data in coated seeds with the highest percentage (177.72%) being recorded in response to Nano 1 mg/ml. For uncoated seeds of the selected plant species, there was a fluctuation in the shoot-root ratio. Overall The impact of various seed priming techniques with three distinct soaking periods (4, 8, and 12 h) on the seedling vigor index (SVI) was shown for coated and uncoated seeds of *P. maritimum* grown in pots. The results were displayed in Fig. (6i). TheSVI showed a significant increase in response to the Nano 1 mg/ml treatment, compared to the control. It was observed that SVI trends increased noticeably after the uncoated seeds of the selected plant species were soaked for three various periods of soaking.

Several seed priming methods were found to cause significantly increasing trends in the biomass of the coated seeds of selected plant species when soaking coated seeds. The soaking of the coated seeds for 8 h increases the biomass significantly. the treatment with the following concentrations of Nano (0.1, 0.5 and 1 mg/ml) has a significant impact on biomass. They resulted in a remarkable increase of 0.31%, 0.53%, and 1.64%, respectively, when compared to the control. After soaking uncoated seeds of a selected plant species for 8 h using various priming methods, a significant trend in data was observed in biomass. The highest increase was achieved with Nano 0.5 mg/ml which showed a considerable percentage increase of 1.26% compared to the control. When uncoated seeds were soaked for 8 h, a significant increase in biomass was observed in response to Nano 0.1, 0.5 mg/ml treatments 1.51% and 1.61%, respectively compared to the control. After soaking uncoated seeds of a selected plant species for 12 h, a significant increase in biomass was observed in response to Nano 0.1 and 0.5 mg/ml treatments by 0.82 and 0.59, respectively compared to the control.

### Chemical analysis

The data of alkaloids represented in Table (2) using GC-MS analysis of *P*. *maritimum* with different nanopriming treatments showed that the quantity of alkaloids decreased in all treatments except in Nano 0.5 mg/ml soaked in 4 h it increased 120% as compared to control. Table (3) shows the data of pancratistatin in *P. maritimum* with different nanopriming treatments in different soaking durations the data recorded that there is no significant difference in the amount of pancratistatin due to different treatments. The result of GC-MS analysis of *P*. *maritimum* recorded a significant increase in Lycorine in treatment compared to control, the highest increase (140%, 184.6% and 163.7%) recorded in 1 (mg/ml) Nano at 4, 8, 12 h soaking duration respectively (Table 4). Antioxidant content was recorded in Table (5). The data of GC-MS analysis of *P*. *maritimum* recorded a significant increase in Antioxidant in nanopriming treatment compared to control, the highest increase (156.9%, 186.18% and 167%) recorded in 1 mg/ml Nano at 4, 8, 12 h soaking duration respectively.

**Table 2:** Mean values of alkaloids content (%) (mean ± S.D., *n* = 3) of *Pancratium maritimum* L. in response to different concentration of nanopriming under different soaking durations

Alkaloids	4 h	8 h	12 h	F	*P*
Control	13.54^bc^ ± 1.74	13.54^a^ ± 1.74	13.54^b^ ± 1.74	–	–
Nano 0.1 (mg/ml)	9.45^deB^ ± 0.42	12.47^abA^ ± 0.48	13.46^bA^ ± 0.54	55.936^*^	< 0.001^*^
Nano 0.5 (mg/ml)	16.34^aA^ ± 0.53	9.87^cdB^ ± 0.86	10.47^deB^ ± 0.39	98.518^*^	< 0.001^*^
Nano 1 (mg/ml)	8.69^eA^ ± 0.66	9.87^cdA^ ± 0.73	9.65^deA^ ± 0.33	3.318	0.107
F	28.091^*^	24.978^*^	42.253^*^		
p_0_	< 0.001^*^	< 0.001^*^	< 0.001^*^		

**Table 3:** Mean values of Pancratistatin content (%) (mean ± S.D., *n* = 3) of *Pancratium maritimum* L. in response to different concentration of nanopriming under different soaking durations

Pancratistatin	4 h	8 h	12 h	F	*P*
Control	2.13^a^ ± 0.22	2.13^a^ ± 0.22	2.13^ab^ ± 0.22	–	–
Nano 0.1 (mg/ml)	2.14^aA^ ± 0.05	2.15^aA^ ± 0.05	1.98^abA^ ± 0.27	1.029	0.413
Nano 0.5 (mg/ml)	2.19^aA^ ± 0.06	1.95^abA^ ± 0.19	1.87^abA^ ± 0.17	3.671	0.091
Nano 1 (mg/ml)	2.10^aA^ ± 0.10	1.84^abA^ ± 0.0	2.16^abA^ ± 0.21	4.717	0.059
F	1.908	4.116^*^	3.596^*^		
p_0_	0.121	0.006^*^	0.011^*^		

**Table 4:** Mean values of Lycorine content (%) (mean ± S.D., *n* = 3) of *Pancratium maritimum* L. in response to different concentration of nanopriming under different soaking durations

Lycorine	4 h	8 h	12 h	F	*P*
Control	29.45^e^ ± 1.22	29.45^f^ ± 1.22	29.45^e^ ± 1.22	–	–
Nano 0.1 (mg/ml)	29.64^deB^ ± 0.37	29.47^fB^ ± 0.39	46.74^abA^ ± 0.50	1669.25^*^	< 0.001^*^
Nano 0.5 (mg/ml)	38.46^bA^ ± 0.52	36.95^dB^ ± 0.48	36.45^dB^ ± 0.18	18.595^*^	0.003^*^
Nano 1 (mg/ml)	41.36^aC^ ± 0.36	54.36^aA^ ± 0.18	48.21^aB^ ± 0.32	1458.31^*^	< 0.001^*^
F	120.911^*^	745.604^*^	712.552^*^		
**p**_**0**_	< 0.001^*^	< 0.001^*^	< 0.001^*^		

**Table 5:** Mean values of Antioxidant content (%) (mean ± S.D., *n* = 3) of *Pancratium maritimum* L. in response to different concentration of nanopriming under different soaking durations

Antioxidant	4	8	12	F	*P*
Control	9.12^f^ ± 0.27	9.12^e^ ± 0.27	9.12^d^ ± 0.27	–	–
Nano 0.1 (mg/ml)	12.14^bcB^ ± 0.32	12.64^bcAB^ ± 0.44	13.24^bA^ ± 0.19	8.356^*^	0.018^*^
Nano 0.5 (mg/ml)	12.89^bA^ ± 0.35	11.67^dB^ ± 0.22	11.58^cB^ ± 0.32	17.549^*^	0.003^*^
Nano 1 (mg/ml)	14.31^aC^ ± 0.28	16.98^aA^ ± 0.53	15.24^aB^ ± 0.22	40.550^*^	< 0.001^*^
F	75.785^*^	150.746^*^	130.155^*^		
p_0_	< 0.001^*^	< 0.001^*^	< 0.001^*^		

SD: **Standard deviation**

**F**: **F for One-way ANOVA test** pairwise comparison bet. each 2 groups were done using a **Post Hoc Test (Tukey)**

p: p-value for comparing between the three different studied time

p_0_: p-value for comparing the different studied groups

*: Statistically significant at *p* ≤ 0.05

Means in the same **Column** with **any Small Common letter**^**(a−i)**^ are not significant (**OR** Means with **totally Different letters**^**(a−i)**^ are significant)

Means in the same **Row** with **any Capital Common letter**^**(A−C)**^ are not significant (**OR** Means with **totally Different letters**^**(A−C)**^ are significant)

## Discussion

Chitosan nanoparticles have been attracting a lot of interest recently due to their numerous uses in the pharmacological, therapeutic, and agricultural fields. Previous studies showed that chitosan nanoparticles as plant growth promoters were the subject of much investigation [18]. Through the imbibition process, chitosan enters the seeds and, improves the germination index, shortens the time it takes for germination and flowering, increases plant development, and produces more biomass as a result of the interaction between the seed and chitosan [19] [20–22]. came to the following conclusions based on studies on the effects of nanoparticles on the germination mechanism of seeds: they increased the seeds’ absorption of water; they increased the concentration of the enzyme nitrate reductase; they promoted the seed antioxidant system; they reduced antioxidant stress by lowering the levels of H_2_O_2_, superoxide radicals, and malonyldialdehyde content; and they increased the activities of some enzymes, such as superoxide dismutase, ascorbate peroxidase, guaiacol peroxidase, and catalase, which improved seed germination in certain plant species.

Our results revealed that there were no significant differences in (GP%) among nanopriming treatments compared to the control in both coated and uncoated seeds. However, there was a significant difference between either coated and uncoated seeds, as the results indicated that GP% was better in coated seeds. The removal of the seed coat reduced germination rate relative to coated seeds [23]. The seed coat is the primary line of defense against environmental stress and pathogen invasion, and it plays an important role in determining seed vitality and germination [24, 25]. Abdel-Aziz also found that higher concentrations of chitosan nanoparticles adversely affected germination and seedling growth [26]. This effect could be due to stimulating capacity of chitosan nanoparticles metabolic activity, which enhances the intrinsic potential for seed development through the absorption of chitosan nanoparticles through imbibition, coating, or seed priming [27–29].

Our findings demonstrated that applying chitosan nanoparticles boosted plant height and biomass. Farooq et al. showed that seedlings originating from chitosan nanoparticles treated seeds showed a significant increase in root length under normal conditions [30] Choudhary et al. found that the Cu-chitosan nanoparticles seed treatments improved the shoot length and vigor index of maize seedlings under normal conditions. However, there was no significant effect on the germination percentage [31]. These results may be related to earlier studies on chitosan, which showed that it could promote growth by increasing the availability and uptake of water and vital nutrients by altering cell osmotic pressure and decreasing the accumulation of harmful free radicals (ORS) by promoting antioxidant and enzyme activity [32]. In comparison to the control, rice growth was found to be greatly increased by a 40 ppm chitosan solution, according to Chamnanmanoontham et al. [33]. Additionally, compared to the control, Nano improved fresh and dry weight, in addition to increasing seed germination in chickpea [34]. Applications of chitosan boosted the stem and leave dry weight in peppermint [35, 36].

Previous research has suggested that *P. maritimum* bulbs contain alkaloids and flavonoids with potential medicinal uses [1–3, 37]. Among those alkaloids is pancratistatin, which exhibits anticancer effects [38]. Analgesic, antimalarial, purgative, acaricidal, insecticidal, anti-migratory, antiviral, antimicrobial, antifungal, immunostimulant, anticancer and antioxidant properties are all present in *P*. *maritimum* extract. More recently, certain research has shown that the alkaloids urngimionorine, pancratistatin, and lycorine have strong anticancer properties [3, 37].

The use of chitosan nanoparticles can induce metabolic processes by increasing the content of bioactive compounds. These compounds play a prominent role in the development and stimulation of secondary metabolite production [39–41]. Our data showed that the quantity of alkaloids decreased in all treatments except in Nano 0.5 (mg/ml) soaked in 4 h it increased compared to the control. the data recorded that there is no significant variance in the amount of pancratistatin due to different treatments. However, a significant increase in Lycorine was observed in the treatment groups compared to the control. The various ways in which chitosan’s chemically and natively synthesized constituents function could alter the physicochemical status of medicinal plants by acting as strong elicitors and biostimulants [42–44]. it enhances the production of secondary metabolites, it may also offer photoprotection. Through the regulation of nitric oxide and hydrogen peroxide signaling, they have a significant role in the reversal of oxidative damage. Additionally, they maintain ion homeostasis, increases photosynthesis by interacting with abscisic acid-induced stomatal closure, improves the activity of antioxidant enzymes including catalase, superoxide, dismutase and peroxidase at the cellular level, and generally enhances growth performance [45–47].

Antioxidant activities increased overall across different nanopriming concentrations and priming broad beans with Nano could increase antioxidant enzymes and total phenolic accumulation, giving seeds more resistant to oxidative stress [26]. This could indicate that the very low quantity of Nano improved seed defenses by raising total phenols and antioxidant enzyme activity. It can significantly reduce plant oxidative stress and increase agricultural output [48, 49], and exposure to chitosan boosts antioxidant and defense enzyme activity A chitosan application increased the formation of amarogentin, mangiferin, and swertiamarin from shoot cultures of swertia (*Swertia paniculata* Wallich), which have strong bioactive qualities that make them very valuable in the pharmaceutical industry [50].

## Conclusion

The application of chitosan nanopriming in seeds holds significant promise as an innovative and efficient approach to enhance seed performance, germination, and overall plant growth. The findings indicated that soaking for 8 h in Nano (1 mg/ml) produced the best production of some compounds such as Pancratistatin and Lycorine. By creating favorable conditions for plant growth and development, nanopriming may contribute to enhanced synthesis of secondary metabolites with medicinal properties.

## Data Availability

All data generated or analysed during this study are included in this published article.

## Abbreviations

GP%: Germination percentage
CVG: The coefficient of Velocity of Germination
SG: Speed of germination
GE: Germination energy
GI: Germination index
MGT: Mean germination time
SVI: Seed vigor index
SL: Seedling shoot length
RL: Seedling root lengt
SE: Standard error
RSA: Radical scavenging activity
WC: Water content
